# Cerebral cysticercosis mimicking subarachnoid hemorrhage: a case report

**DOI:** 10.1186/s41016-021-00258-w

**Published:** 2021-09-02

**Authors:** Tao Liu, Tingzhong Wang, Yijun Bao, Jinghua Du, Yongchang Guan

**Affiliations:** 1 Department of Neurosurgery, The Fourth Affiliated Hospital of China Medical University, Shenyang, 110032 China; 2 Department of Neurology, The Fourth Affiliated Hospital of China Medical University, Shenyang, 110032 China

**Keywords:** Cerebral cysticercosis, Subarachnoid hemorrhage, Computed tomography, Magnetic resonance imaging, Misdiagnosis

## Abstract

**Background:**

Dense exudate during the calcification of cerebral cysticercosis in basal subarachnoid space was easy to be misdiagnosed as subarachnoid hemorrhage (SAH); clinical evaluation and MRI can help differentiate SAH from pseudo-SAH.

**Case presentation:**

A case of ventricular expansion accompanied by high-density shadows in cisterna circinata cerebri was taken to the hospital for treatment due to sudden faint. This patient was diagnosed as subarachnoid hemorrhage according to computed tomography (CT) in another hospital. We believe that the high density in cisterna circinata cerebri was misdiagnosed as subarachnoid hemorrhage (SAH) 1 year ago. The main etiology of SAH is aneurysm; non-aneurysmal SAH associated with cerebral cysticercosis is extremely rare. Only 5 patients have been reported.

**Conclusion:**

This case indicated that although the specificity of CT for SAH is very high, the physicians should be aware of rare false positive findings, called pseudo-SAH.

**Supplementary Information:**

The online version contains supplementary material available at 10.1186/s41016-021-00258-w.

## Background

Cerebral cysticercosis is the most common parasitic diseases caused by the larval stage of *Taenia solium*, involving central nervous system (CNS) [1]. This tapeworm is endemic in most developing countries where pigs are raised, and continues to be one of the most important causes of seizures in the world [2]. Although *Taenia solium* infection is not endemic in the USA, many neurocysticercosis cases are found in the immigrants from endemic areas of Asia, Africa, Eastern Europe, Mexico, and South America [3]. With increases in immigration from endemic regions, numbers of patients with neurocysticercosis are increasing in the USA. Both adult worm and larvae can infect human, and the larvae gain easily access to brain, eye, and subcutaneous tissue through the small bowel wall to the lymphatics or circulatory system [4, 5]. It has been found that more than 60% patients with cerebral cysticercosis can show clinical symptoms after 7 years infection [6]. According to different location of the cysts, cerebral cysticercosis has four main forms: meningeal, parenchymal, ventricular, and mixed lesions. The form of meningeal can be further divided into two subgroups, namely, dorsolateral subarachnoid space and basal subarachnoid space. Cerebral cysticercosis presents protean manifestations including seizures, signs of elevated intracranial pressure (ICP) depending on the number of parasites, different locations, and the degree of host immunologic response to infestation [7].

In this study, we present the case of a man with subarachnoid form of cerebral cysticercosis and his dense exudate of cerebral cysticercosis in basal subarachnoid space was misdiagnosed as subarachnoid hemorrhage. Then, we review the pertinent literature on the subject to better this uncommon entity.

## Case presentation

A 49-year-old male with a sudden-onset loss of consciousness, presented to our emergency department (ED), with 1-year history of headache, nausea, vomiting, weakness in the limbs, thirst with desire of drink, hoarseness, and dysphagia.

The patient had similar symptoms 1 year ago, including nausea and vomiting. Head CT were performed in a local hospital (Fig. S1A). Cisterna circinata cerebri with high density image was revealed, and Pandy test in cerebrospinal fluid (CSF) was positive. Then, he was diagnosed as subarachnoid hemorrhage and received relevant symptomatic treatment. But further examination showed that there were no red blood cells in his cerebrospinal fluid, and CTA (Fig. S1B) indicated his intracranial arterial system was normal. Because of indifference and there was no obvious improvement, the patient discharged 3 days later.

**Fig. 1:**
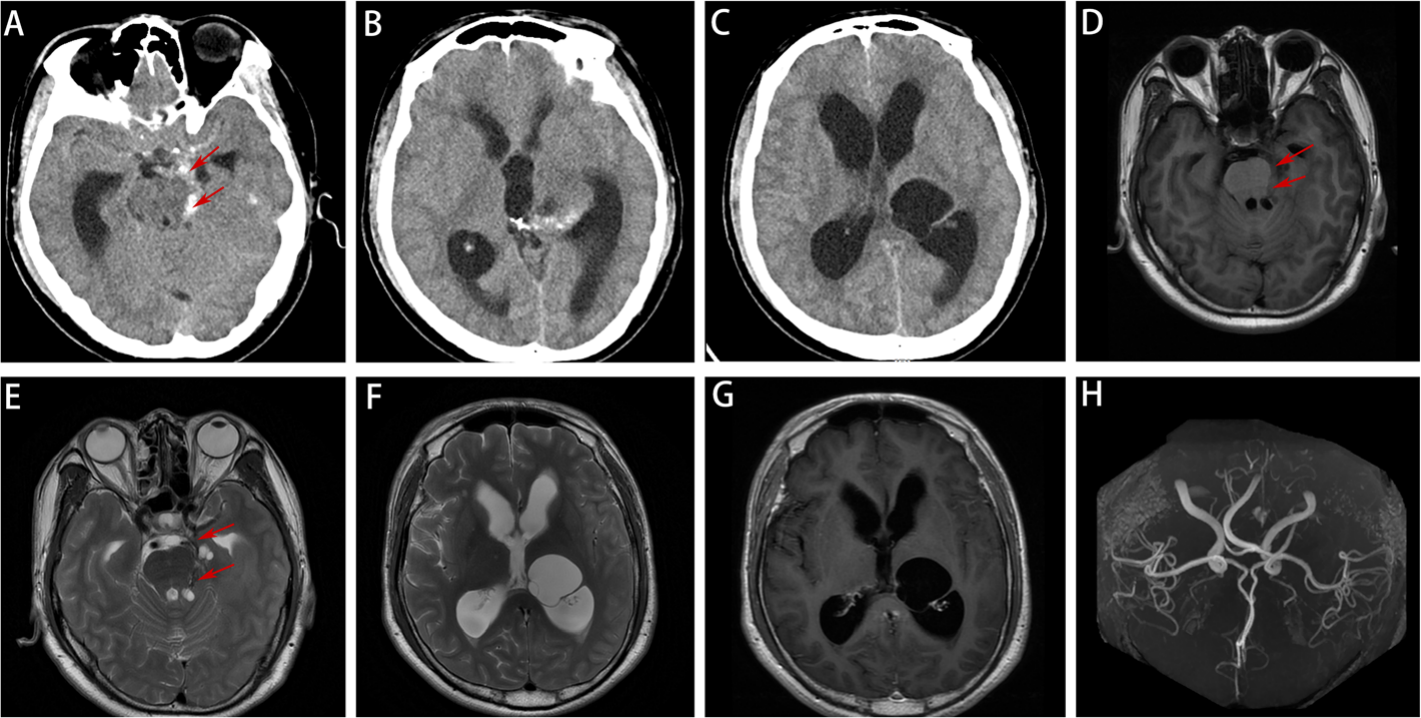
CT scan images showed high-density shadows in cisterna circinata cerebri were seen (**A**), and bilateral lateral ventricle and third ventricle dilatation (**B**). Multiple cystic lesions were seen in the third ventricle, the left basal ganglia region, the left hippocampal region (**C**). MRI results showed that T1 and T2 presented low signal (**D**, **E**). T2 presented high signal in the third ventricle, the left basal ganglia region, the left hippocampal region (**F**), with no significant enhancement after contrast-enhanced scanning (**G**). Furthermore, MRA indicated no aneurysms found in intracranial arterial system (**H**)

After the patient was referred to our hospital, he had a Glasgow Coma Scale score of 15 with retrograde amnesia, there was no fever, pupils were isochoric and normally reactive to light. No obvious abnormality was observed in respiratory, cardiovascular, and gastrointestinal systems. Limb muscle strength was level 4, muscle tension was normal, limbs sophisticated activities were weaken. Physiological reflex was normal, and pathological reflex including meningeal signs was negative (Fig. 1).

The patient had a history of cysticercosis 30 years ago, and the CT value of the high-density shadow near the thalamus and cisternae measured 92 Hu without perihematomal edema, so we considered the high-density shadow as calcification. During the subsequent treatment, there was no significant change in the density (Fig. S2), which also proved our opinion. According to these findings, we thought that the diagnosis was cerebral cysticercosis. Therefore, we performed antibody detection of cysticercosis and found IgG was positive.

At admission, this patient treated with albendazole (15 mg/kg/day); meanwhile, short course of dexamethasone (20 mg/day) along with fatty meals promotes drug absorption [8]. In first 3 days, the patient had a worse headache and dizziness, according to known side effects of albendazole, we reviewed the patient’s head CT, and monitored blood counts and liver enzymes but there is nothing unusual.

After regular treatment, the patient had less headache and nausea and no other discomfort; we performed the CT again a month later. CT scan images indicated that the left basal ganglia cystic lesion was significantly smaller and hydrocephalus was slightly smaller than before, there were calcifications in the cystic lesions indicating that the treatment was effective (Fig. S2). Because of financial reasons, at day 30 post-treatment in the hospital, the patient required to be conservative treated at home. Upon evaluation of the patient, we found he was asymptomatic. CSF analysis revealed 10 cells/mm^3^ (monocyte 15%, neutrophils 6%), 158 mg/dL protein, and 2.8 mg/dL glucose. Enzyme-linked immunosorbent assay test on CSF for cerebral cysticercosis was negative.

One month later after discharge, the patient became sluggish, no dizziness, headache, nausea, and vomiting. The patient was referred to hospital again, CT scan images showed obvious dilation of bilateral ventricle and third ventricle (Fig. S3B), high density shadow was seen in left cerebral cistern and thalamus area (Fig. S3A). At admission, we performed antibody detection of cysticercosis and found IgG was negative. After a consult, the patient had a ventriculoperitoneal shunt placed because of the obstructive hydrocephalus. Postoperatively, cefodizime sodium (1 g) was administrated intravenously twice a day for 3 days from postoperative day 1 followed by mannitol (50 g) intravenously once a day for 3 days. On the third day after the operation, the signs of hydrocephalus were better than before, and the clinical symptoms were less. No obvious discomfort was observed after drug withdrawal. The patient was discharged on postoperative day 7, the patient came to the hospital for re-examination 2 week after hospital discharge, and CT showed that supratentorial hydrocephalus was significantly better than before (Fig. S3C, S3D); meanwhile, the patient showed no symptoms of sluggish with a normal gait.

## Discussion and conclusions

Cysticercosis is common in developing countries. Reports from China demonstrate prevalence rates of 0.06%, which may climb to 9.83% in some provinces. It was estimated that around 0.37 million people with cysticercosis live in China [9]. Meanwhile, the relationship between cerebral cysticercosis and stroke is well established previously. The incidence of stroke in neurocysticercosis varies from 2.0 to 11.8% [4, 10]. It has been mostly reported that cerebral cysticercosis is associated with ischemic, hemorrhagic strokes mainly referring to SAH were relatively rare. SAH have been noted in subarachnoid cerebral cysticercosis and have been associated with cerebral aneurysms in some cases [11–15]. The main etiology of SAH is aneurysm, non-aneurysmal SAH associated with cerebral cysticercosis is extremely rare. Only 5 patients have been reported in Table 1.

**Table 1:** Non-aneurysmal SAH caused by cerebral cysticercosis

Report	Sex/age	Clinical symptoms	Imaging finding	CSF analysis	Medication	Outcome
1. Sawhney et al. 1998 [16]	M/10y	Headache, nausea, vomiting, and partial seizures	Left parietal SAH Multiple cysts	NA	Albendazole steroids	No improvement
2. Tellez-Zenteno et al. 2003 [17]	F/32y	Headache, dysarthria, hemiparesis, psychomotoragitation	SAH	WBC: 3/ml Glucose: 55 mg/dL Proteins: 33 mg/dL	Albendazole steroids	Improvement
3. Tellez-Zenteno et al. 2003 [17]	M/34y	Headache, nausea, vomiting, diplopia, gait disorder, psychomotor agitation, CNS impairment	SAH	WBC: 107/ml (97%L) Glucose: 19 mg/dL Proteins: 695 mg/dL	Albendazole steroids	Improvement
4. Viola et al. 2011 [12]	F/39y	severe headache	SAH around the left Sylvian fissure	RBC: 18/ml (78% L, 5% E) WBC: 20/ml Glucose: 88 mg/dL protein: 28 mg/dL	Dexamethasone Albendazole	Fully recovered
5. Cardenas et al.	F/38y	headache ,vomiting , blurred vision. Meningeal signs and papilledema	SAH Hydrocephalus Cysts	WBC: 147/ml (86%L) Glucose: 1 mg/dL Proteins: 222 mg/dL	Prednisone albendazole	Improvement
6. This study	M/49y	Headache, nausea, vomiting, weakness in the limb, hoarseness, dysphagia	Hydrocephalus Cysts	WBC: 592/ml (67%L) RBC: 0/ml Glucose: 30 mg/dL Proteins: 178 mg/dL	Dexamethasone Albendazole	Improvement

*NA* information not available, *CSF* cerebrospinal fluid, *L* lymphocytes, *E* eosinophils

Among the 5 included case, the ages ranged from 10 to 39 (30.6 ± 11.8). Three patients (60%) were female, two patients (40%) were male, headache predominated in 5 (100%) and blurred vision in 2 (40%) patients. Other symptoms mainly were movement disorder, nausea, and vomiting. Most patients presented with epileptic seizures [18, 19], but our case did not. A review showed that over 50% of patients with subarachnoid cerebral cysticercosis have imaging evidence of vasculitis [20]. But in our patient, no one finding in neuro-imaging studies was the presence of inflammatory aneurysm. Observation to be seen clearly is that patients with non-aneurysmal SAH associated with cerebral cysticercosis mainly predominates in younger adults having fewer classic cardiovascular risk factors compared to patients with stroke non-associated with cerebral cysticercosis. In clinical practice, aneurysms are considered first in young patients with subarachnoid hemorrhage. That is indisputable. Because of aneurysmal SAH is a subtype of nontraumatic cerebrovascular disease, which account for 3% to 5% of strokes and approximately 85% of the subarachnoid hemorrhage [21].

As described previously, in the early stage of cerebral cysticercosis, a large number of larvas enter the systemic circulation of host, causing widespread infection of brain tissue along with high fever, headache, and increased intracranial pressure. Nevertheless, at this time, the brain CT lack of typical imaging changes. As we all know, two types of cysts are inclined to develop in the brain tissue: cysticercus cellulosae and cysticercus racemosus.

Cysticercus racemosus, varying in size from 4-12 cm, tends to grow in the basal subarachnoid spaces and induce inflammatory comprising chronic meningitis. The pathogenesis of inflammatory in neurocysticercosis is not clear. Cysts will shrink in 2-5 years and form calcium deposits which could block third ventricle, the fourth ventricle, and midbrain aqueduct causing hydrocephalus. Cysts in basal subarachnoid space produce dense exudate and eventually form calcification. The density of dense exudate in the intermediate stage is lower than that of calcification, but higher than that of normal brain tissue and cerebrospinal fluid. The authors think that it is likely to be close to the density of acute hemorrhage. Because these exudates are in subarachnoid space, they are likely to be misdiagnosed as subarachnoid hemorrhage.

Strict inclusion criteria were used in the literature review, those did not report sufficient information to categorize them as aneurysmal or non-aneurysmal would be excluded. The literatures indicated that the non-aneurysmal SAH in these five cases was caused by cerebral cysticercosis [12, 14, 16, 17]. Magnetic resonance imaging (MRI) or cerebral angiograms did not show any evidence of aneurysms or vascular malformations. So, we classify them as non-aneurysmal. All cases of SAH were confirmed by CT, the misdiagnosis experience of our case gave us a reflection on whether there were misdiagnoses of SAH in the above five cases. Then, we turned our attention to CSF analysis, CSF examination is a reliable auxiliary method for the diagnosis of SAH, especially for suspected SAH, but only a case showed the number of red blood cells in the CSF increased [12]; information of other cases about red blood cells was not available. Therefore, we believe that the four cases of SAH were suspicious [14, 16, 17] and more evidence is needed to further confirm.

There may be several reasons for the misdiagnosis: Firstly, the patient is young without cardiovascular risk factors, accompanied by headache, nausea, and vomiting. Combined with the imaging findings of the patient, it is easy to be confused with aneurysmal SAH. Second, when the larva gain easily access to brain, the density of dense exudate in the intermediate stage is lower than that of calcification, but higher than that of normal brain tissue and cerebrospinal fluid; there might be meningitis in initial stage, for example, Pandy test in CSF was positive, confirmatory evidence for cerebral cysticercosis is difficult to find. Finally, comprehensive analysis ability of physician is low, and the medical history is not careful enough to obtain; this patient had a history of cysticercosis 30 years ago, there is no relevant description of medical history in the patient’s medical record.

In conclusion, cerebral cysticercosis is an important cause of neurological diseases that spread in endemic areas. The presentation of the disease is pleomorphic and requires a combination of clinical evaluation, imaging, and laboratory investigation. Furthermore, patients with cysticercosis must be systematically treated to avoid recurrence.

## Supplementary Information

**Additional file 1.** Fig. S1. Radiologic images obtained by CT scan one year ago. Fig. S2. Radiologic images obtained by CT scan at day-30 post-treatment. Fig. S3. Radiologic images obtained by CT scan at day-60 (A, B) and day-74 post-treatment.

## Data Availability

Data sharing is not applicable to this article as no datasets were generated or analyzed during the current study.

## Abbreviations

SAH: Subarachnoid hemorrhage
CT: Computed tomography
MRI: Magnetic resonance imaging
CNS: Central nervous system
ICP: Intracranial pressure
ED: Emergency department
CSF: Cerebrospinal fluid
