# Supplementary material for: Cerebral cysticercosis mimicking subarachnoid hemorrhage: a case report
Source: Chin Neurosurg J. 2021 Sep 2;7:39. doi: 10.1186/s41016-021-00258-w (PMC8411545; doi:10.1186/s41016-021-00258-w)

**Supplementary material**

**Fig.S1.** Radiologic images obtained by CT scan one year ago

**Fig.S2.** Radiologic images obtained by CT scan at day-30 post-treatment

**Fig.S3.** Radiologic images obtained by CT scan at day-60 (A, B) and day-74 post-treatment

**Fig.S1. Radiologic images obtained by CT scan one year ago.** **A:** CT scan image showed cisterna circinata cerebri presents a high density shadow (red arrow in **A**). **B:** CAT scan image showed that the vascular morphology of the intracranial arterial system was normal.

**
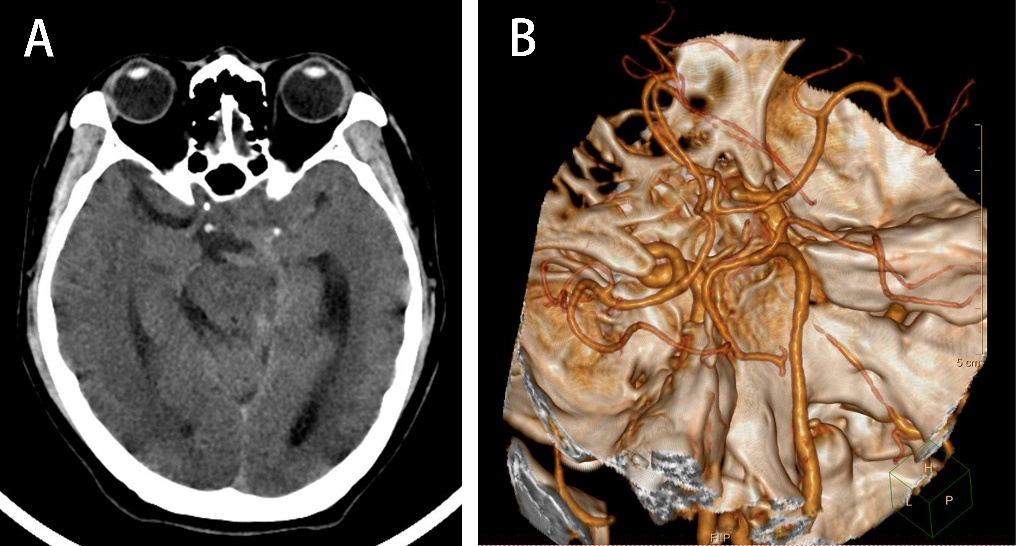
**

**Fig.S2.** **Radiologic images obtained by CT scan at day-30 post-treatment.** **A:** CT scan image showed that the calcification was seen near the left midbrain without obvious change compared to the image taken before treatment (**Fig.S2A**). **B:** CT scan image showed that hydrocephalus was less than before (**Fig. 2B**).

**
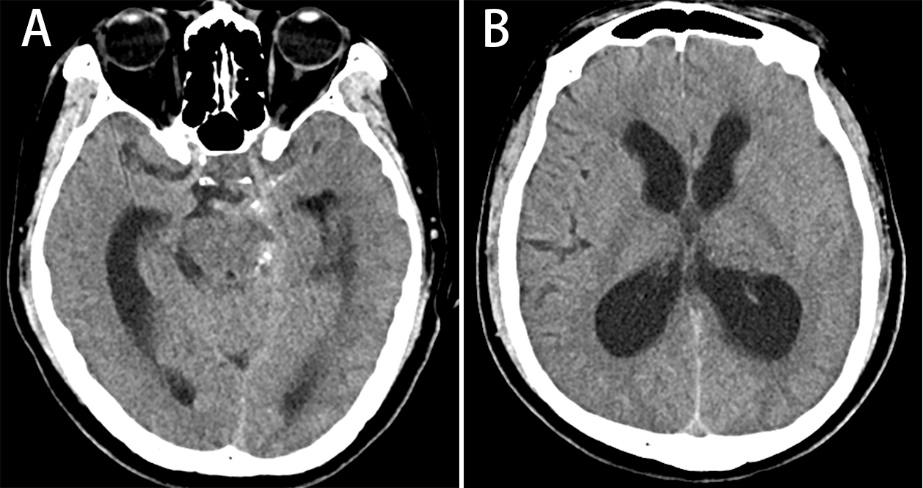
**

**Fig.S3. Radiologic images obtained by CT scan at day-60 (A, B) and day-74 post-treatment (C, D).** Calcification is seen near the left cisterns after 60 days treatment (**A**), bilateral ventricles, and the third ventricle is significantly dilated (**B**). High density calcification shadows were seen in the thalamus region (**B**). After ventriculoperitoneal shunt, signs of hydrocephalus were reduced after 10 weeks treatment (**C, D**).


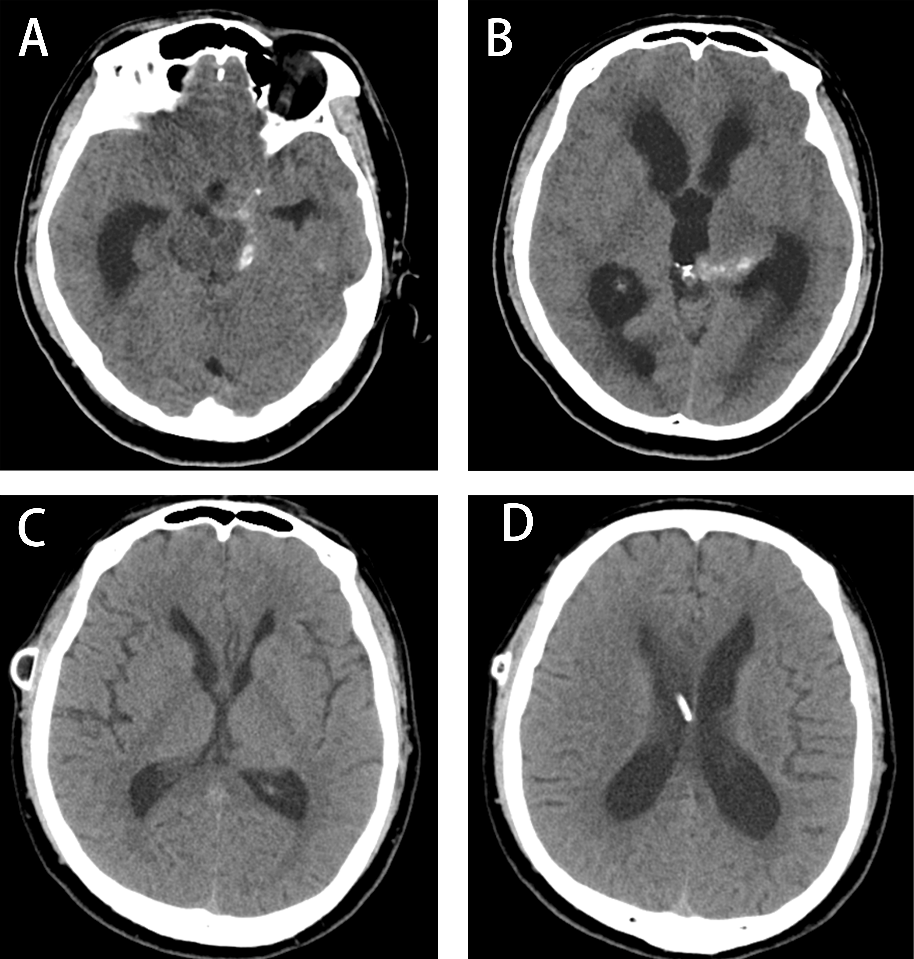

Supplement: Supplementary file 1 — Additional file 1. Fig. S1. Radiologic images obtained by CT scan one year ago. Fig. S2. Radiologic images obtained by CT scan at day-30 post-treatment. Fig. S3. Radiologic images obtained by CT scan at day-60 (A, B) and day-74 post-treatment. [file 41016_2021_258_MOESM1_ESM.docx]
